# Workplace violence against hospital personnel in Tunisia: prevalence, associated psychological and behavioral outcomes, and legal actions

**DOI:** 10.3389/fpubh.2026.1894676

**Published:** 2026-09-07

**Authors:** Zeineb Mallek, Hanen Maamri, Mouna Baklouti, Emna Mziou, Maissa Ben Jmeaa, Maroua Chaaben, Imen Sboui, Raouf Karray, Jihen Jdidi, Sourour Yaich

**Affiliations:** 1 Department of Preventive and Community Medicine, Hedi Chaker University Hospital, Sfax, Tunisia; 2 Department of Preventive and Community Medicine, Faculty of Medicine of Sfax, Sfax, Tunisia

**Keywords:** health personnel, litigation, mental health, Tunisia, workplace violence

## Abstract

**Background:**

Workplace violence against hospital personnel is a significant occupational and public health concern that can compromise care quality and staff wellbeing. This study aimed to estimate its prevalence, describe its forms and outcomes, and identify associated factors, legal actions, and psychological outcomes.

**Methods:**

A cross-sectional study was conducted over 4 months (August–November 2024) in the Hedi Chaker University Hospital and Habib Bourguiba University Hospital in Sfax. A representative sample of hospital personnel completed an anonymous self-administered questionnaire.

**Results:**

A total of 406 hospital personnel were included. The sex ratio (M/F) was 0.24, and the median age was 36 years (IQR: 30–45). The prevalence of verbal, physical, and sexual violence was 63.1% (*n* = 256), 17% (*n* = 69), and 2% (*n* = 8), respectively. Legal action was taken by 24 participants (9.6%). Multivariate analysis revealed that verbal violence was independently associated with overcrowded workplace (AOR = 0.12; *p* = 0.002) and poor medical services and a lack of hospital supplies (AOR = 7.3; *p* = 0.007), while physical violence was associated with patient death (AOR = 2.6; *p* = 0.003) and age ≥40 years (AOR = 1.7; *p* = 0.042). Taking legal action was more frequent after physical violence (COR = 8.08; *p* < 0.001) and among personnel working in intensive care units (COR = 14; *p* = 0.02), and less frequent among physicians (COR = 0.2; *p* = 0.042). Physical violence was significantly associated with insecurity, hypervigilance, mental health consultation for anxiety, depressive, and post-traumatic stress-related symptoms, social impairment, and sleep disorders (all *p* < 0.05).

**Conclusion:**

Workplace violence in Sfax university hospitals was frequent and strongly associated with psychological outcomes, highlighting the need to strengthen prevention strategies, improve communication and training, and ensure adequate psychological support for hospital personnel.

## 1 Introduction

Workplace Violence (WPV) is a major public health issue affecting all professional sectors worldwide, regardless of the economic context. The International Labor Organization (ILO) defines this phenomenon as any unacceptable behavior, practice, or threat that could result in physical, psychological, sexual, or economic harm to workers (1). This violence can take many forms, ranging from verbal to physical violence, and affects a significant proportion of employees. This phenomenon is particularly important in the healthcare sector, where close, repeated contact with the public, often in stressful, urgent, or distressing situations, is associated with a higher risk of violence against hospital personnel. According to the World Health Organization (WHO), nearly 62% of hospital personnel have faced WPV (2). WPV is regarded as one of the most complex occupational hazards that hospital personnel encounter (3). Certain specialties, particularly psychiatry and emergency departments, are characterized by higher levels of WPV (4, 5). The study of Iozzino et al. has shown that approximately one in five patients hospitalized in acute psychiatric units commits at least one act of violence during hospitalization, exposing hospital personnel to a substantial risk of aggression (6). Overall, WPV can have profound consequences for hospital personnel’s health and wellbeing. The consequences of WPV are not limited to physical harm. They are accompanied by significant psychological repercussions, such as anxiety, depression, burnout, demotivation, and even dismissal from work (7, 8). These effects directly affect the quality of patient care, alter the caregiver-patient relationship, and reduce the efficiency of hospital departments. In addition to its physical and psychological consequences, WPV can lead hospital personnel to take legal action against perpetrators. Legal action is intended not only to hold those responsible accountable, but also to enhance security within the hospitals. The decision to take legal actions may depend on several factors, such as the knowledge of legal rights, perceived support from the organization, and potential barriers, such as fear of retaliation or stigmatization. Understanding these factors is essential to encourage reporting and ensure adequate protection for hospital personnel. At the international level, WPV against hospital personnel has been widely documented in various contexts, particularly in Europe (4), Asia (9), and Africa (10). However, in Tunisia, research on WPV among hospital personnel remains limited (11, 12). Few studies have been conducted about its prevalence and consequences for hospital personnel. Therefore, to fill this knowledge gap, we conducted this study, aiming to assess the prevalence of WPV among hospital personnel, characterize its different forms, explore factors associated with both its occurrence and with taking legal actions, and evaluate differences in psychological and behavioral outcomes according to the type of violence.

## 2 Materials and methods

### 2.1 Study design and setting

This was a cross-sectional study conducted from August to November 2024 among a sample of hospital personnel working in two university hospitals in Sfax, Southern Tunisia: One primarily provides medical care, and the other primarily provides surgical care.

### 2.2 Study population

The study included hospital personnel (medical, paramedical, administrative staff, and non-healthcare workers) working in the two university hospitals of Sfax during the survey period who provided informed consent. The category “non-healthcare workers” refers to non-clinical support staff, including cleaning, maintenance, logistics, and transport personnel, who are not classified as administrative, medical, or paramedical staff. Hospital personnel absent during the study period (annual leave, secondment, or training), and unusable questionnaires were excluded. The minimum required sample size was estimated at 379 participants using a 5% alpha risk, 5% precision, and an expected prevalence of workplace violence of 56.3%, based on a previous 2019 study conducted among nurses in two university hospitals in Sfax (12).

Data were collected using a stratified random sampling method with proportional allocation at two levels. The overall required sample size of 379 participants was first distributed between the two hospitals in proportion to the total number of hospital personnel employed at each institution, rather than being divided equally. Within each hospital, an exhaustive list of hospital personnel was subsequently established according to professional category, and the hospital-specific sample was further allocated proportionally across these categories based on their relative size, as detailed in Table 1. A simple random draw was then performed within each professional category at each hospital until the required number of participants per stratum was reached. To anticipate potential non-responses while preserving statistical power and proportional balance across all strata, a total of 419 questionnaires (representing the minimum sample size increased by approximately 10%) were distributed across all subgroups according to the initial sampling frame. Of these, 10 individuals (2.4%) refused to participate, and 3 questionnaires (0.7%) were excluded due to being unusable. Non-responders were not replaced. Ultimately, 406 participants were included in the final analysis, successfully exceeding the minimum required sample size. This surplus of 27 participants over the minimum target was distributed proportionally across all hospital and professional categories, thereby preserving the exact stratified allocation ratios established in the initial sampling frame. This yielded a high final response rate of 96.9% (406/419). The final sample maintains optimal representativeness relative to the target study population. Data collection was carried out using a self-administered, anonymous, and structured questionnaire. This questionnaire was adapted from several validated instruments found in the literature (13), notably that conducted jointly by the WHO and the ILO on violence in the workplace in the health sector (14). To ensure linguistic and cultural appropriateness for the local context, the original survey instruments underwent forward translation into French, followed by back-translation into English to ensure semantic and conceptual equivalence. The questionnaire was administered in French; while the socio-demographic and occupational characteristics section was presented strictly in French, all subsequent sections systematically included Arabic translations in parentheses alongside the French items to facilitate comprehension across all staff categories. Before the main survey, a pilot study was conducted among 15 hospital personnel (who were excluded from the final analysis) to assess question clarity, comprehension, and completion time. A panel of occupational health experts reviewed the face and content validity. The complete questionnaire is provided as Supplementary material S1.

**Table 1:** Minimum required sample size by university hospital center and professional category.

Professional category	Hedi Chaker university hospital total (%)	Sample	Habib Bourguiba university hospital total (%)	Sample
Total	2033 (50.8)	193	1972 (49.2)	186
Administrative staff	125 (6.1)	12	77 (3.9)	7
Physicians	237 (11.6)	23	269 (13.6)	26
Paramedical staff	840 (41.3)	80	985 (49.9)	93
Non-healthcare workers	409 (20.1)	38	336 (17.1)	32
Interns	149 (7.3)	14	65 (3.3)	6
Residents	273 (13.4)	26	240 (12.2)	22

%: Percentage. “Non-healthcare workers” = non-clinical support staff (cleaning, maintenance, logistics, and transport personnel).

**Figure 1:**
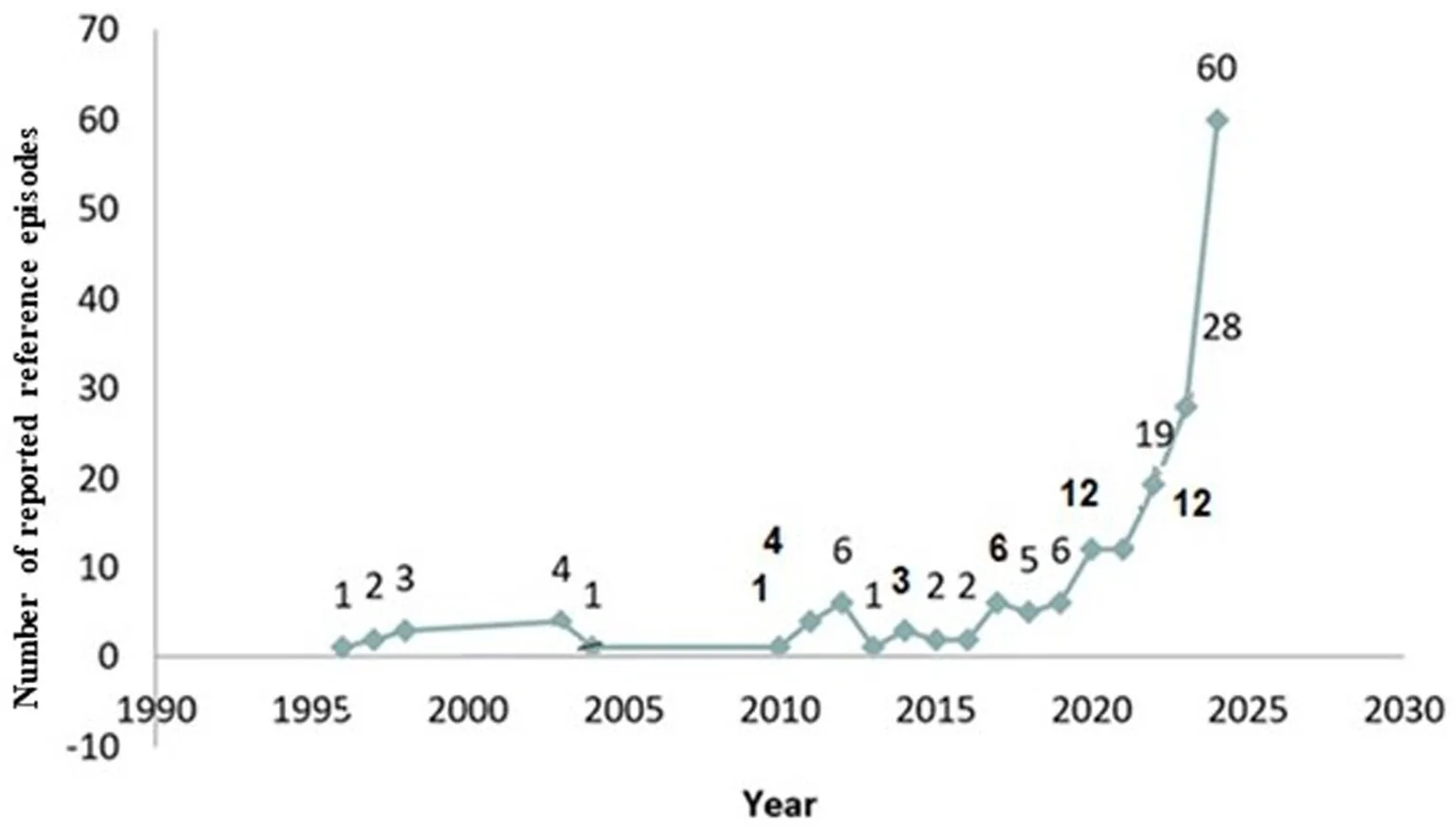
Distribution of the year of the most shocking violence episode reported by participants (1996–2024). A single reported reference episode per participant (the most shocking one).

The questionnaire was divided into five sections. The first section focused on individual characteristics (age, gender, education level) and professional characteristics (seniority, position held). The second section addressed the prevalence of violence, including its types and forms. The third section examined the reasons for violent incidents, while the fourth section explored their psychological and behavioral outcomes. Post-traumatic stress, anxiety, and depressive symptoms were considered present when participants reported having consulted a mental health professional specifically because of the violent incident. This pragmatic, self-reported indicator was used instead of validated psychometric scales because the questionnaire already covered multiple domains, and the inclusion of comprehensive diagnostic instruments would have considerably increased its length, potentially reducing participation and response quality. The fifth section was devoted to the possible legal actions taken by hospital personnel. Prevalence estimates were calculated on a per-person basis (lifetime prevalence), corresponding to the proportion of hospital personnel reporting at least one episode of violence; participants with multiple episodes were counted only once. For the chronological analysis, participants who specified a year reported the year of their most shocking episode of violence; Figure 1 thus reflects the distribution of a single reference episode per participant, rather than the cumulative number of incidents.

### 2.3 Statistical analysis

Qualitative variables were described using frequencies and percentages. Quantitative variables were presented as means and standard deviations when their distribution followed a normal distribution. In the absence of normality, they were described using medians and interquartile ranges (IQR). The normality of quantitative variables was assessed using the Shapiro–Wilk and Kolmogorov–Smirnov tests. For each dependent variable—including physical violence, sexual violence, legal actions, and various psychological outcomes- associated factors were analyzed using hypothesis tests. Proportions were compared using Pearson’s χ^2^ test when the conditions for its application were met, and Fisher’s exact test was used when these conditions were not met. Multivariate analysis was performed using binary logistic regression to identify factors independently associated with verbal violence exclusively (defined as verbal violence occurring without concomitant physical or sexual violence), and physical violence. Due to the limited number of outcome events, legal action was excluded from multivariable modeling to avoid overfitting and sparse data bias. For each type of violence evaluated, two separate multivariable models were constructed to accommodate structural differences in the applicable populations. The first model focused on individual and professional factors and was applied to the entire study sample. The second model investigated the perceived reasons for violence; this analysis was inherently restricted to the subsample of participants who reported experiencing at least one form of WPV. Merging these variables into a single model was avoided to prevent the unjustified exclusion of non-victims from the analysis of individual and professional factors. Variables with a significance level of less than 0.20 in univariate analysis were included in the respective models, allowing mutual control for potential confounding among these factors. No additional variables with *p* ≥ 0.20 were forced into the multivariable models, as none were identified as mandatory clinical or epidemiological confounders. All eligible variables were entered simultaneously into the multivariable model using the Enter method. Multicollinearity among independent variables was assessed before model fitting using the Variance Inflation Factor (VIF). Cases with missing data for variables included in a given model were excluded from that analysis (complete-case analysis).

For each factor entered into the multivariable model, the adjusted odds ratio (AOR), 95% confidence interval (95% CI), and *p*-value were calculated and reported completely in the results tables to demonstrate all adjusted estimates.

The quality of the final models was assessed using overall classification accuracy and the Hosmer-Lemeshow test, which assesses the agreement between the observed values and those predicted by the model. Model discrimination was evaluated using the Area Under the Receiver Operating Characteristic (ROC) Curve (AUC). For the analysis of psychological and behavioral outcomes, each outcome was analyzed individually as a binary dependent variable in univariable analysis in relation to each type of violence experienced, without multivariable adjustment. These analyses were restricted to participants who reported experiencing WPV and completed the psychological and behavioral outcomes section. Given the large number of pairwise comparisons and the small cell sizes for certain exposure subgroups (e.g., sexual violence), these unadjusted analyses were considered exploratory. A *p*-value of less than 0.05 was considered statistically significant.

### 2.4 Ethical considerations

The study was conducted in strict compliance with ethical research principles. The anonymity and confidentiality of participants’ data were guaranteed. Informed consent was obtained from each professional before their participation. Additionally, the agreements of the hospital directors of both university hospitals were obtained. This study was also approved by the Ethics Committee of the Faculty of Medicine of Sfax under the number 19/26.

## 3 Results

### 3.1 Sociodemographic and professional characteristics

Our study included 406 hospital personnel working in both hospitals. The sex ratio (M/F) was 0.24. Their median age was 36 years [Interquartile Range (IQR) = (30–45) years]. Regarding their occupations, 45.3% (*n* = 184) were paramedics, while 18.5% (*n* = 75) were non-healthcare workers (Table 2).

**Table 2:** Socio-demographic and professional characteristics of hospital personnel and workplace violence exposure.

Characteristics	Number (N)	Percentage (%)
Gender		
Male	79	19.5
Female	327	80.5
Educational level		
Primary	35	8.6
Secondary	43	10.6
University	328	80.8
Marital status		
Married	282	69.5
Unmarried, divorced, widowed	124	30.5
Chronic disease	100	24.6
Profession		
Paramedical staff (nurses, nursing assistants, technicians)	20	45.3
Administrative	75	4.9
Non- healthcare workers	51	18.5
Senior doctors	51	12.6
Medical resident	184	12.6
Medical intern	25	6.2
Job position		
Permanent hospital personnel	302	74.4
Temporary hospital personnel	98	24.1
Substitute hospital personnel	6	1.5
Work seniority		
≤10	248	61.1
>10	158	38.9
Department		
Medical	164	40.4
Surgical	136	33.5
Administration/laboratory	70	17.2
Intensive care unit	36	8.9
Work time		
Morning	400	98.5
Afternoon	159	39.2
Nightshift	124	30.5
Verbal violence	256	63.1
Forms of verbal violence (*n* = 256)		
Impolite manner of conversation	232	90.6
Shout/scream	235	91.8
Verbal threat	143	55.9
Insult	163	63.7
Interruption /not listening	160	62.5
Racial harassment	48	18.8
Physical violence	69	16.99
Forms of physical violence (*n* = 69)		
Push with hands or kick	62	89.9
Objects thrown	14	20.3
Armed assault	7	10.1
Sexual violence	8	2
Forms of sexual violence (*n* = 8)		
Harassment	1	12.5
Intimidation	4	50
Unwanted sexual behavior	5	62.5
Indecent exposure	6	75
Sexual assault	1	12.5

Percentages for subtypes are calculated among victims of the corresponding violence type (verbal, *n* = 256; physical, *n* = 69; sexual, *n* = 8). Subtypes are indented under their parent category.

### 3.2 Prevalence and forms of workplace violence

When asked about their exposure to violence, 63.1% (*n* = 256) reported having experienced at least one form of workplace violence—verbal, physical, and/or sexual—at least once during their career, with a confidence Interval (CI 95%) = (58.6–67.7%). Physical violence and sexual violence were reported at rates of 16.99% [*n* = 69; CI95% = (13.5–20.9%)] and 2% [*n* = 8; CI95% (0.7–3.4%)], respectively (Table 2). The prevalence of any WPV (63.1%) was identical to that of verbal violence because every participant who experienced physical or sexual violence had also experienced verbal violence. Specifically, 45.6% [*n* = 185/406; 95% CI (40.4–50.5%)] of participants experienced verbal violence exclusively. Regarding the forms of violence, shouting or screaming and impolite manners of conversation were the most reported forms of verbal violence (91.8%, *n* = 235) and (90.6%, *n* = 232), respectively. Pushing with hands was the most cited form of physical violence (89.9%, *n* = 62). Regarding sexual violence, unwanted sexual behavior and indecent exposure were the most frequently reported forms (62.5%, *n* = 5 and 75%, *n* = 6, respectively), while sexual assault and harassment were each reported by only one participant (12.5%) (Table 2).

Among hospital personnel, 178 (69.5%) specified the year in which the most shocking episode of workplace violence occurred. The most frequently reported year was 2024 (*n* = 60; 33.7%) (Figure 1).

### 3.3 Factors associated with workplace violence

Regarding verbal violence, univariate analysis showed no significant association with any individual or professional factor. However, multivariate analysis revealed that poor medical services, a lack of hospital supplies (AOR = 7.3; *p* = 0.007), and overcrowded workplaces (AOR = 0.12; *p* = 0.002) were independently linked to verbal violence. As for physical violence, Multivariate analysis revealed that patient death (adjusted odds ratio (AOR) = 2.6; *p* = 0.003) and age ≥ 40 years (AOR = 1.7, *p* = 0.042) were independently associated with physical violence. However, no significant difference was found between sexual WPV and individual/professional factors or the perceived reasons of violence (Table 3).

**Table 3:** Association between type of workplace violence and individual/professional factors and perceived reasons for violence among hospital personnel.

Factor	Verbal violence exclusively (*n* = 185)				Physical violence (*n* = 69)				Sexual violence (*n* = 8)	
Individual and professional factors (sample = 406)										
	COR* [95% CI**]	p***	AOR**** [95%CI]	*p*	COR* [95% CI**]	p***	AOR**** [95%CI]	*p*	COR [95% CI]	*p*
Gender										
Male	1	0.322			1	0.418			1	0.68
Female	1.28 [0.78–2.12]				1.33 [0.66–2.67]				0.72 [0.14–3.63]	
Age										0.49
<40 years	1	0.57			1	***0.004*** ^†^	1	**0.042** ^†^	1	
≥40 years	1.13 [0.74–1.72]				1.64 [1.2–2.78]		1.7[1.1–3.1]		0.57 [0.11–2,88]	
Work seniority										
<10 years	1	0.79			1	***0.041*** ^†^	1	0.3	1	0.73
≥ 10 years	1.06 [0.7–1.59]				1.63 [1.1–2.758]		1.4 [0.6–3.1]		0.77[0.18–3.29]	
Job position										
Temporary/substitute	1	0.29			1	0.68			1	0.16
Permanent	0.77 [0.49–1.25]				0.89 [0.49–1.59]				0.2[0.05–0.85]	
Occupation										
Administrative	1	0.93			1	0.38			1	0.96
Paramedical	1.22 [0.47–3.13]	0.68			1.2 [0.3–4]	0.7			–	–
Worker	1.12 [0.41–3.07]	0.83			1.7 [0.9–3.2]	0.08			–	–
Medical	1.06 [0.41–2.78]	0.9			1.4 [0.6–3]	0.35			–	–
Department										
Administration/laboratories	1	0.09			1	***0.04*** ^†^	1	0.3	–	0.226
Intensive care units	0.69 [0.28–1.67]	0.42			0.9[0.2–3.1]	0.9	0.7 [0.1–4.1]	0.6	–	–
Surgical	1.72 [0.92–3.19]	0.088			2.5[1.1–5.1]	***0.019*** ^†^	2.1 [0.7–5.8]	0.14	–	–
Medical	1.43 [0.79–2.58]	0.24			1.6[0.7–3.7]	0.2	1.7 [0.6–4.5]	0.29	–	–
Reasons for violence (sample = 256)										
Patient impatience	0.4 [0.09–2.04]	0.28			1.02 [0.54–1.94]	0.93			0.31 [0.07–1.31]	0.097
Dissatisfaction with the care provided	0.5 [0.09–2.04]	0.78			1.1 [0.63–1.91]	0.72			0.58 [0.13–2.48]	0.45
Long waiting time	0.6 [0.2–1.8]	0.38			1.38 [0.79–2.4]	0.25			0.61 [0.14–2.60]	0.5
Insufficient staff	0.4 [0.14–1.3]	0.18	0.4 [0.1–1.3]		1.13 [0.65–1.96]	0.65			1.80 [0.42–7.72]	0.41
Overcrowded workplace	0.2 [0.05–0.7]	***0.014*** ^†^	0.12 [0.035–0.48]	***0.002*** ^†^	1.44 [0.83–2.52]	0.18	1.1 [0.5–2.1]	0.9	0.67 [0.15–2.87]	0.58
Workload	0.5 [0.18–1.5]	0.29			0.97 [0.56–1.7]	0.94			2.05 [0.48–8.79]	0.32
Poor medical services and a lack of hospital supplies	5.01 [1.1–22]	***0.028*** ^†^	7.3 [1.7–30.1]	***0.007*** ^†^	1.01 [0.57–1.77]	0.96			1.4 [0.34–5.75]	0.63
Patient death	0.4 [0.15–1.4]	0.18	0.6 [0.18–2.2]		2.55 [1.34–4.85]	***0.004*** ^†^	2,6 [1,3–5,2]	***0.003*** ^†^	0.56 [0.06–4.70]	0.59
Low level of education and aggressive attitude	0.8 [0.2–2.5]	0.7			1.49 [0.82–2.70]	0.17	1.53 [0.8–2.8]	0.17	0.58 [0.14–2.37]	0.44

COR*, Crude Odds Ratio; 95%CI**, 95% Confidence Interval; p***, *p* value; AOR****, Adjusted Odds Ratio Binary logistic regression (Enter method; candidate variables with *p* < 0.20). • Verbal violence exclusively (Reasons model, *n* = 256; 185 events): Hosmer–Lemeshow Chi-square = 0.17 (*p* = 0.800); classification accuracy = 72.1%; AUC = 0.773 (95% CI: 0.654–0.892). No model was fitted for individual/professional characteristics as no variable met the entry criteria. • Physical violence (Individual/Professional model, *n* = 406; 69 events): Hosmer–Lemeshow Chi-square = 13.2 (*p* = 0.519); classification accuracy = 83.0%; AUC = 0.670 (95% CI: 0.541–0.720). • Physical violence (Reasons model, *n* = 256; 69 events): Hosmer–Lemeshow Chi-square = 1.60 (*p* = 0.446); classification accuracy = 71.9%; AUC = 0.633 (95% CI: 0.550–0.711). No additional variables with *p* ≥ 0.20 were forced into the multivariable models, as none were identified as mandatory clinical or epidemiological confounders. ^†^indicates *p* < 0.05 statistically significant.

### 3.4 Association between WPV exposure and its psychological and behavioral outcomes

Among the 256 participants who reported experiencing at least one form of violence, 213 (83.2%) completed the psychological outcomes assessment and were included in the outcome analyses. Physical violence was significantly associated with several psychological, behavioral, and professional outcomes. Victims were more likely to feel insecure at work (COR = 2.94, *p* = 0.018), to remain on high alert (COR = 2.35, *p* = 0.015), to repeatedly think about the violent incident (COR = 4.42, *p* < 0.001), to perceive that all tasks required great effort (COR = 2.56, *p* = 0.004), to report sleep disorders (COR = 4.12, *p* < 0.001), and to experience an impact on social life (COR = 2.08, *p* = 0.019). They were also more likely to seek mental health consultation for anxiety-related symptoms (COR = 3.48, *p* < 0.001), for post-traumatic stress-related symptoms (COR = 3.65, *p* < 0.001), and for depressive symptoms (COR = 2.92, *p* = 0.001). In terms of professional consequences, they more frequently considered changing their workplace (COR = 2.17, *p* = 0.016) or their career (COR = 1.92, *p* = 0.04), and reported absence from work (COR = 3.22, *p* = 0.007) (Table 4). In contrast, no significant association was found between sexual violence and any of the psychological or behavioral outcomes studied (all *p* > 0.05) (Table 4).

**Table 4:** Association between exposure to workplace violence and its psychological and behavioral outcomes among hospital personnel.

Outcomes	Type of violence	Physical violence				Sexual violence			
		No (*n* = 144)	Yes (*n* = 69)	COR* [95% CI**]	p***	No (*n* = 206)	Yes (*n* = 7)	COR [95% CI]	*p*
Feeling insecure	No	37 (25.7)	7 (10.5)	1	***0.018*** ^†^	41 (19.9)	3 (42.9)	1	0.159
	Yes	107 (74.3)	62 (89.5)	2.94 [1.17–7.41]		165 (80.1)	4 (57.1)	0.34[0.07–1.61]	
Being on high alert	No	59 (41)	16 (22.8)	1	***0.015*** ^†^	74 (35.9)	1 (14.3)	1	0.226
	Yes	85 (59)	53 (77.2)	2.35[1.16–4.74]		132 (64.1)	6 (85.7)	3.46[0.41–29.35]	
Sadness	No	59 (41)	18 (26.3)	1	0.052	73 (35.4)	4 (57.1)	1	0.256
	Yes	85 (59)	51 (73.7)	1.94[0.98–3.82]		133 (64.6)	3 (42.9)	0.42[0.092–1.946]	
Thinking about changing the workplace	No	75 (52.1)	23 (33.3)	1	*0.016*	95 (46.1)	3 (42.9)	1	0.833
	Yes	69 (47.9)	46 (66.7)	2.17[1.14–4.12]		111 (53.9)	4 (57.1)	1.17[0.25–5.4]	
Perceiving that all the tasks require a great effort	No	78 (54.2)	22 (31.6)	1	*0.004*	97 (47.1)	3 (42.9)	1	0.791
	Yes	66 (45.8)	47 (68.4)	2.56[1.34–4.89]		109 (52.9)	4 (57.1)	1.22[0.26–5.63]	
Mental health consultation for anxiety-related symptoms	No	86 (59.7)	21 (29.8)	1	*<0.001*	104 (50.4)	3 (42.9)	1	0.651
	Yes	58 (40.3)	48 (70.2)	3.48[1.81–6.73]		102 (49.6)	4 (57.1)	1.42[0.309–6.506]	
Repeatedly thinking about the violent incident	No	94 (65.3)	21 (29.8)	1	*<0.001*	111 (53.8)	4 (57.1)	1	0.917
	Yes	50 (34.7)	48 (70.2)	4.42[2.27–8.58]		95 (46.2)	3 (42.9)	0.92[0.2–4.23]	
Mental health consultation for post-traumatic stress-related symptoms	No	98 (68.1)	25 (36.8)	1	*<0.001*	119 (57.7)	4 (57.1)	1	0.91
	Yes	46 (31.9)	44 (63.2)	3.65[1.92–6.94]		87 (42.3)	3 (42.9)	1.09[0.24–5.012]	
Planning to immigrate	No	92 (63.9)	34 (49.1)	1	0.054	122 (59.2)	4 (57.1)	1	0.88
	Yes	52 (36.1)	35 (50.9)	1.83[0.98–3.41]		84 (40.8)	3 (42.9)	1.11[0.24–5.12]	
Impact on social life	No	94 (65.3)	33 (47.4)	1	*0.019*	121 (58.7)	6 (85.7)	1	0.160
	Yes	50 (34.7)	36 (52.6)	2.08[1.12–3.89]		85 (41.3)	1 (14.3)	0.243[0.03–2.05]	
Mental health consultation for depressive symptoms	No	98 (68.1)	29 (42.1)	1	*0.001*	123 (59.7)	4 (57.1)	1	0.845
	Yes	46 (31.9)	40 (57.9)	2.92[1.55–5.51]		83 (40.3)	3 (42.9)	1.16[0.25–5.34]	
Negative outlook on the future	No	94 (65.3)	36 (52.6)	1	0.096	125 (60.7)	5 (71.4)	1	0.590
	Yes	50 (34.7)	33 (47.4)	1.69[0.91–3.15]		81 (39.3)	2 (28.6)	0.63[0.12–3.35]	
Thinking of a career change	No	98 (68.1)	36 (52.6)	1	*0.04*	129 (62.6)	5 (71.4)	1	0.664
	Yes	46 (31.9)	33 (47.4)	1.92[1.02–3.59]		77 (37.4)	2 (28.6)	0.69[0.13–3.66]	
Sleep disorders	No	106 (73.6)	28 (40.4)	1	*<0.001*	131 (63.6)	3 (42.9)	1	0.231
	Yes	38 (26.4)	41 (59.6)	4.12[2.16–7.86]		75 (36.4)	4 (57.1)	2.47[0.54–11.36]	
Impact on relations with colleagues	No	106 (73.6)	46 (66.7)	1	0.325	146 (70.9)	6 (85.7)	1	0.4
	Yes	38 (26.4)	23 (33.3)	1.39[0.72–2.71]		60 (29.1)	1 (14.3)	0.41[0.05–3.49]	
Doubt about performance	No	127 (88.2)	54 (78.9)	1	0.093	174 (84.5)	7 (100)	1	0.269
	Yes	17 (11.8)	15 (21.1)	1.99[0.88–4.49]		32 (15.5)	0 (0)	NE****	
Work absence	No	133 (92.4)	54 (78.9)	1	*0.007*	182 (88.3)	5 (71.4)	1	0.147
	Yes	11 (7.6)	15 (21.1)	3.22[1.33–7.81]		24 (11.7)	2 (28.6)	3.29[0.60–18.06]	

Of the 256 participants reporting violence, 213 completed the psychological outcomes section (missing data excluded 1 sexually exposed participant, leaving 7/8 in these analyses). COR*, Crude Odds Ratio; 95%CI**, 95% Confidence Interval; p***, *p-*value; NE****, Not estimable Odds Ratio not estimable owing to a zero cell. These univariate analyses are exploratory; given the number of comparisons, no adjustment for multiple testing was applied. ^†^indicates *p* < 0.05 statistically significant.

### 3.5 Factors associated with taking legal actions among hospital personnel

Legal action was taken by 9.6% of hospital personnel who had been victims of WPV (*n* = 24). Higher odds of taking legal action were observed among health personnel aged 40 years or older (COR = 4.00; *p* = 0.001), those with 10 years or more of work seniority (COR = 4.42; *p* = 0.001), permanent staff (COR = 4.70; *p* = 0.023), support workers (COR = 1.50; *p* = 0.047), personnel working in intensive care units compared with administration/laboratory (COR = 14.00; *p* = 0.020), and victims of physical violence (COR = 8.08; *p* < 0.001). In contrast, belonging to the medical profession was associated with lower odds of taking legal action compared with administrative staff (COR = 0.20; *p* = 0.042) (Table 5).

**Table 5:** Factors associated with taking legal action among hospital personnel: univariate analysis.

Factors		Univariate analysis		
		Yes *n* (%)	COR* [95% CI**]	p***
Age	<40 years	8 (5.1)	1	***0.001*** ^†^
	≥ 40 years	16 (17.6)	4 [1.64–9.77]	
Gender	Male	5 (11.1)	1	0.712
	Female	19 (9.3)	0.82[0.29–2.33]	
Profession	Administrative	2 (16.7)	1	***0.005*** ^†^
	Paramedical	9 (8)	0.4 [0.1–2.2]	0.3
	Worker	11 (23.9)	1.5 [1.1–8.1]	***0.047*** ^†^
	Medical	2 (2.6)	0.2 [0.1–2.2]	***0.042*** ^†^
Work seniority	<10 years	6 (4.3)	1	***0.001*** ^†^
	≥10 years	18 (16.5)	4.42[1.69–11.55]	
Department	Administration/laboratory	1 (2.4)	1	***0.04*** ^†^
	Intensive care unit	5 (26.3)	14 [1.5–73.3]	***0.02*** ^†^
	Surgical	7 (7.4)	3.2 [0.3–23.2]	0.2
	Medical	11 (11.6)	5.2 [0.6–43.2]	0.1
Job position	Temporary/substitute	2(2.9)	1	***0.023*** ^†^
	Permanent	22(12.3)	4.7[1.1–20.8]	
Physical violence	No	7 (3.9)	1	***<0.001*** ^†^
	Yes	17 (24.6)	8.08 [3.18–20.54]	
Sexual violence	No	23 (9.5)	1	0.78
	Yes	1 (12.5)	1.35[0.16–11.49]	

COR*, Crude Odds Ratio; 95%CI**, 95% Confidence Interval; p***, *p*-value. For categorical variables with more than two categories, the *p*-value on the reference row indicates the overall association with the outcome; *p*-values on other rows indicate comparisons with the reference category. ^†^indicates *p* < 0.05 statistically significant.

## 4 Discussion

### 4.1 Prevalence and forms of workplace violence

The results of our study showed that nearly two-thirds of hospital personnel (63.1%) had experienced at least one form of violence during their professional career. A study conducted in Sfax, Tunisia, in 2019 at the same two public hospitals indicated a lower WPV of 56.3% (12). Although the independent cross-sectional design of the two surveys prevents us from confirming a temporal increase, the marked difference observed within the same setting is noteworthy. This finding deserves attention, especially in the context of persistent staff shortages. Among the different forms of violence, verbal violence was the most common, reflecting a high prevalence comparable to that reported in other studies of hospital professionals (15, 16). Our result was also similar to studies conducted in Ethiopia and Jordan, which reported prevalences of WPV of 64% (17) and 65.5% (18), respectively. However, this prevalence was lower in many studies, including China, which reported a decreased prevalence of WPV among nurses (26.6%) (19). WPV may have been temporarily modulated during the COVID-19 pandemic, when increased public respect and gratitude toward healthcare professionals could have attenuated certain forms of violence in some settings (20). However, it is still uncertain whether this decline continued after the pandemic, as some studies have reported that workplace violence returned to pre-pandemic levels in certain settings. Physical violence, although less frequent (17%), remains a cause for concern and is consistent with the range reported by the WHO, which estimated a prevalence between 8 and 38% (21).

The data analysis revealed that most participants who specified a year situated their most shocking episode in 2024 33.7% The distribution of reference episodes across calendar years should be interpreted cautiously. It may have been influenced by the relatively short professional experience of the participants, with 61.1% having less than 10 years of service, as well as by the greater salience of recent events when recalling the most shocking episode (10, 22).

Of the three forms of WPV reported, verbal violence emerged as the most frequent, affecting 63.1% of the respondents (*n* = 256) and was higher than physical violence (16.99%, *n* = 69), which was consistent with many studies (23–25). This may be because verbal violence remains difficult to prevent, as it often occurs suddenly and cannot be stopped by security measures alone (26). In addition, this may be explained in part by its ease of access for the aggressor, its perceived lower penalty, and the stress or frustration of patients and their companions.

In addition, the prevalence of sexual violence was equal to 1.97% (*n* = 8), which was inconsistent with certain international studies where its prevalence is higher (15). Indeed, in an umbrella review of 14 meta-analyses including 674,266 Healthcare professionals worldwide, the pooled prevalence of sexual harassment was 10.5% (27). Similarly, a meta-analysis of 47 studies among Chinese healthcare professionals estimated the prevalence of sexual violence at 6.3% (28). Estimates reach even higher levels in certain populations, such as a two-center Greek study where 70.3% of surveyed nurses and physicians indicated experiencing sexual harassment during their professional career (29). This low prevalence may be explained by the fact that hospital personnel underreport being exposed to sexual violence due to cultural reasons, social stigma, and fear of potential repercussions (30). Moreover, methodological heterogeneity may partly account for this variability. A recent systematic review reported prevalence estimates of sexual violence against health personnel ranging from 0 to 68%, reflecting the lack of a standardized definition and the frequent inclusion of sexual violence within broader measures of WPV (31).

### 4.2 Factors associated with workplace violence

Unlike physical violence, exposure to verbal violence was not significantly associated with any individual sociodemographic or professional characteristic, suggesting that verbal violence in the two hospitals is a widespread phenomenon affecting hospital personnel regardless of their gender, age, occupation, or department. Instead, only environmental and organizational factors remained independently associated with verbal violence. In fact, poor medical services and a lack of hospital supplies were independently associated with verbal violence. A recent literature review highlighted that insufficient resources and staffing relative to patient demand, long wait times, and poor organizational support were the main factors contributing to violence against hospital personnel (32). Similarly, in an Egyptian study conducted in the emergency departments of Ain Shams University Hospitals, a lack of resources was the risk factor most frequently reported by hospital personnel who were victims of violence (33). Unexpectedly, hospital personnel working in overcrowded departments were less likely to experience verbal violence. One possible explanation is that these departments are often staffed by larger teams, enabling colleagues to support one another and collectively manage difficult situations. The constant presence of multiple hospital personnel may also help discourage aggressive behavior from patients or their relatives and facilitate the early de-escalation of conflicts. However, this finding differs from those of most previous studies, which have consistently identified overcrowding as a risk factor for WPV (34, 35). Therefore, it should be interpreted with caution, as it may reflect organizational characteristics specific to the hospitals included in this study.

Regarding factors associated with WPV, age equal to or greater than 40 was significantly associated with physical violence. This finding is in agreement with the study conducted by Ouma et al. in Morocco, which observed that nurses in the 40–49 and ≥ 60 age groups showed a significantly positive association with physical violence after standardized residual analysis (36). At the same time, in a study conducted in Ethiopia, authors reported that nurses aged 18–39 were significantly less likely to be exposed to WPV than their colleagues aged 40–55. This difference was explained by the perceived greater efficiency of younger hospital personnel in patient care, which would reduce situations likely to lead to violence (37). Additionally, this could be explained by the fact that the most experienced hospital personnel have accumulated more years of practice and hold positions with greater clinical responsibilities, placing them more frequently in direct contact with patients and their companions in high-tension situations. Moreover, older hospital personnel have had more years at risk because exposure to violence was measured over the course of a professional career, which mechanically raises the cumulative probability of having experienced at least one violent accident.

Contrary to our findings, the study conducted in Abha (Saudi Arabia) did not reveal any significant differences in exposure to violence based on age (38). Furthermore, patient death was significantly associated with physical violence. This result suggests that situations involving a life-threatening prognosis or imminent death may increase stress and frustration in patients or their companions, leading to an increased risk of physical aggression. This highlights the importance of considering clinical crises as high-risk contexts for violence and suggests the need to implement specific prevention and support strategies for hospital personnel exposed to these situations.

As for sexual violence, no factors were identified as being significantly associated with sexual violence. This result could be explained mainly by the small number of hospital personnel who reported having been victims, which limits the statistical power of the analyses and reduces the ability to identify potential associations. These results highlight the need for further studies involving larger samples to explore the determinants of sexual violence in hospitals in greater depth and assess its association with outcomes among hospital personnel.

### 4.3 Psychological outcomes of workplace violence

Additionally, physical violence was significantly associated with several psychological, behavioral, and professional consequences. Victims were more likely to feel insecure at work (COR = 2.94, *p* = 0.018), to remain on high alert (COR = 2.35, *p* = 0.015), to report sleep disorders (COR = 4.12, *p* < 0.001), and to consider changing the workplace (COR = 2.17, *p* = 0.016). They were also more likely to seek mental health consultation for anxiety-related symptoms (COR = 3.48, *p* < 0.001), post-traumatic symptoms (COR = 3.65, *p* < 0.001), and depressive symptoms (COR = 2.92, *p* = 0.001). These results can be explained by the fact that physical violence is a direct traumatic experience that may be associated with prolonged activation of the stress system and a sense of permanent vulnerability. A study on the impact of WPV on nurses’ mental health found that exposed hospital personnel had significantly higher levels of psychological distress. This association was significant for exposure to violence in general and even more pronounced for exposure to physical violence (12). A meta-analysis conducted among nurses showed that exposure to WPV was associated with a significantly higher risk of developing post-traumatic stress disorder or burnout syndrome, compared to professionals who were not exposed (39). Similarly, Zheng et al. reported that exposure to physical violence was a significant risk factor for psychological distress. Hospital personnel who were victims of violence had an increased risk of insomnia (22%), depression (27.4%), and anxiety (31.6%) compared to the unexposed hospital personnel (40).

### 4.4 Factors associated with taking legal action among hospital personnel

In exploratory univariate analysis, several socio-demographic, occupational, and violence-related factors were significantly associated with taking legal action following workplace violence (*n* = 24). Specifically, significantly higher odds of taking legal action were observed among personnel aged 40 years or older (COR = 4.00; *p* = 0.001), those with 10 years or more of work seniority (COR = 4.42; *p* = 0.001), permanent staff (COR = 4.70; *p* = 0.023), support workers (COR = 1.50; *p* = 0.047), personnel working in intensive care units (COR = 14.00; *p* = 0.020), and victims of physical violence (COR = 8.08; *p* < 0.001). Conversely, belonging to the medical profession was associated with lower odds of taking legal action compared to administrative staff (COR = 0.20; *p* = 0.042). The observed associations with older age, greater seniority, and permanent employment status suggest that job stability and professional experience play a decisive role in legal recourse. Senior and tenured staff members may possess greater awareness of their legal rights, benefit from stronger job security, and experience less fear of professional retaliation when initiating formal administrative or legal proceedings (41, 42). Regarding workplace settings, the high likelihood of legal action in intensive care units can be explained.

the specific context of intensive care units, where the severity of clinical situations and high medico-legal stakes encourage a formal response to assaults, particularly physical ones. In fact, when a caregiver is physically assaulted in a high-stakes setting, there is greater motivation, both personal and institutional, to document, report, or take legal action (43). In addition, physical violence, due to its objectifiable nature (injuries, medical certificates, witness statements…), facilitates the creation of a legal case file. In contrast, Physicians may be less likely to take legal action, possibly because of less exposure to certain forms of violence or more frequent underreporting linked to professional status and concerns about reputation or career.

The low rate of taking legal action observed should be understood in light of the prevailing reporting culture in healthcare facilities: WPV is still mostly underreported and often trivialized due to fear of retaliation, unclear procedures, and the complexity of administrative and legal processes (44). In Tunisia, this issue is worsened by the legal framework; in fact, assaults on hospital personnel are covered by general provisions of the Penal Code penalizing violence and contempt of public officials (Articles 125 and 127) (45). However, the lack of a specific law protecting hospital personnel contributes to underreporting and limits the effectiveness of sanctions against such acts of violence. In line with international occupational health frameworks, notably the joint ILO/WHO program on WPV in the health sector, developed with the International Council of Nurses and Public Services International, and ILO Convention No. 190 (1, 14), legal recourse should represent the final step in a comprehensive institutional strategy. Such a strategy should prioritize accessible reporting systems, systematic documentation of violent incidents, and the provision of psychological and legal support for affected hospital personnel.

### 4.5 Strengths and limitations

To the best of our knowledge, this is the first study in Africa to explore the prevalence of legal actions related to WPV. The sample size was sufficient to ensure reliable and representative results. Stratified sampling by professional category, combined with the inclusion of two medical and surgical hospitals, allowed for the coverage of a variety of contexts and ensured internal diversity. In addition, the structured questionnaire, adapted from recognized international references, enabled the collection of relevant data that was comparable to existing literature. Moreover, our study simultaneously explored verbal, physical, and sexual violence, as well as its psychological and behavioral repercussions, and analyzed both the prevalence of legal actions and the factors associated with them. Nevertheless, our study presents certain limitations. The cross-sectional nature of the study does not allow causal relationships to be established between the factors studied and the occurrence of violence or its psychological outcomes. Because exposure was assessed over participants’ entire careers while predictors reflect current characteristics, temporal ambiguity is introduced. Specifically, age and work seniority likely act as proxies for cumulative exposure time, current working conditions may differ from those during past incidents, and perceived organizational stressors risk recall bias or *post-hoc* rationalization. Consequently, identified factors should be interpreted strictly as cross-sectional associations rather than causal risk factors. Furthermore, data collection based on self-reporting may be subject to memory or social desirability biases, as participants may underestimate or overestimate their exposure to violence or its outcomes. However, standardized and anonymous questionnaires were used to minimize these biases and encourage more accurate reporting. In addition, the chronological trend analysis relies on a single reference episode per participant—the most shocking one—rather than on the total number of violent events experienced. While this approach likely limits recall bias, as particularly shocking events tend to remain salient in memory it does not capture the full temporal distribution of violent events and cannot be interpreted as a measure of incidence over time. Moreover, anxiety, depressive, and post-traumatic symptoms were assessed using a pragmatic, self-reported indicator whether the participant had consulted a mental health professional because of the incident rather than validated diagnostic instruments. This may have led to an underestimation of the true prevalence of these symptoms. Additionally, the analysis of psychological and behavioral outcomes was based on univariate comparisons only, without multivariate adjustment, given the small number of exposed participants for sexual violence and the large number of outcomes assessed for physical violence; these findings should therefore be interpreted as hypothesis-generating. Finally, due to the limited number of outcome events for legal actions (*n* = 24), multivariable logistic regression was omitted to avoid overfitting and sparse data bias. Consequently, factors associated with taking legal action were evaluated using exploratory univariate analysis only, and these findings should be interpreted with caution and confirmed with future studies with a larger sample.

## 5 Conclusion

In conclusion, this study highlights the prevalence and complexity of WPV against hospital personnel in Sfax University Hospitals, as well as its significant psychological and professional repercussions. The results underscore the need to strengthen prevention and protection measures, improve the organization of services, clarify procedures for legal actions, and offer psychological support to victims. Staff training and optimizing communication with patients and their families are also key measures. Implementing these integrated strategies would contribute to a safer and more humane working environment, promoting the wellbeing of hospital personnel and the quality of care.

## Data Availability

The original contributions presented in the study are included in the article/Supplementary material, further inquiries can be directed to the corresponding author.
