# Supplementary material for: Workplace violence against hospital personnel in Tunisia: prevalence, associated psychological and behavioral outcomes, and legal actions
Source: Front Public Health. 2026 Sep 7;14:1894676. doi: 10.3389/fpubh.2026.1894676 (PMC13590422; doi:10.3389/fpubh.2026.1894676)
Supplement: Supplementary file 1 [file Supplementary_file_1.DOCX]

**Violence exercée chez les professionnels de la santé en milieu hospitalier**

**العنف المسلط على اعوان الصحة داخل المؤسسات الاستشفائية**

-**Age** :……/**Genre**: Femme	/ Homme

-**Niveau éducationnel** : primaire

/Secondaire	/ universitaire

-**Statut matrimonial** : célibataire	/marié(e)	/ veuf(ve)	divorcé(e)

-**Maladie chronique** : Oui	/	Non

-Si oui, type :…………………………………

-**Hôpital**: Hedi Chaker	/ Habib Bourguiba

**Profession** : Infirmier	/ Aide-soignant	/Ouvrier	/Technicien	/ Administratif

Médecin : Sénior	/résident	/ interne	/

hhorah

Autres :……………………………………

-**Poste** : Permanent	/ temporaire	/ remplacement

-**Service ou unité** : ……………………………

-**Ancienneté dans le poste** : ………en années

-**Horaire** : matinée	/après-midi	/nuit

-**Avez-vous été victime durant votre carrière au moins une fois de violence ?**

oui	/	non

**Si oui : Compléter à répondre :**

-**Nombre de fois :……………………..**

-**Type de violence :**

*Verbale لفظي: oui	/	non

si oui nombre:................................

*Physique جسدي: oui	/	non

si oui nombre:................................

* Sexuelle جنسي: oui	/	non

si oui nombre:................................

*Autre type ……………………………….

**-Fréquence de la violence معدل:**

*Fréquence violence verbale  لفظي par mois (ou par an): …………fois/mois (……/an).

*Fréquence violence physique جسدي par mois (ou par an): …………fois/mois (……/an).

* Fréquence violence sexuelle جنسي par mois (ou par an): …………fois/mois (……/an).

**-En rapport avec l’expérience de violence la plus choquante الأكثر تأثيرا عليك**

-Année de l’accident : …………………

- Année par rapport au début de carrière:…….

- **Si Violence verbale** لفظي**:**

*Conversation impolie قلة احترام: oui	/non

*Crier/hurler صراخ: oui	/non

*Menace verbale تهديد: oui	/	non

*Insultes شتائم: oui	/	non

*Interruption/ne pas écouter مقاطعة: oui	/non

* Harcèlement ethnique et racial عنصرية:

oui	/	/ non

*Autres :…………………………

- **Si** **violence Physique** جسدي**:**

*Pousser avec les mains/coups de pied دفع باليد او الساق: oui	/	non

*Instruments lancés مقذوفات : oui	/	non

*Agression avec armes سلاح  : oui	/	non

*Autres :…………………………

- **Si Violence sexuelle** جنسي **:**

*Harcèlement (verbal)تحرش : oui	/ non

*Intimidation	تخويف : oui	/	non

*Comportement sexuel non désiréايحاءات : oui	/	non

*Exposition indécente التعرض غير اللائق:

oui	/	non

*Agression sexuelle الاعتداء الجنسي: oui	/non

*Autres :…………………………

- **La source de violence** الشخص القائم بالعنف**:**

*Patient مريض : oui	/ non

*Membre de la famille des patients فرد من عائلة المريض: oui	/	non

*Un agent de sécurité أعوان الحراسة:

oui	/ non

*Un collègue زملاء: oui	/ non

*Un supérieur مسؤول: oui	/ non

*Autres……………………………

**-** **La source de violence** الشخص القائم بالعنف**:**

***1) Si patient***  مريض***:***

*Âge du patient (années) :……….

*Durée de l’hospitalisation (jours) : ………..

*Raison de l'admission :……………..

+Maladie aiguë : oui	/	non

+Maladie chronique : oui	/ non

+Opération chirurgicale : oui	/	non

***2) Si supérieur hiérarchique ou collègue :***

*Grade :…………………………..

*Service :…………………………….

- **Les raisons de violence أسباب العنف:**

*Services médicaux médiocres et manque de fournitures hospitalières نقص في الخدمات الصحية: oui	/non

*Personnels insuffisant نقص في الاعوان:

oui	/ non

*Longue durée d’attente  مدة انتظار مرتفعة:

oui	/	non

*Faible niveau d'instruction et attitude agressive الاندفاع و ضعف المستوى التعليمي:

oui	/	non

*Impatience, patient pressé الضغط و عدم التفهم: oui	/ non

*Décès du patient حالة وفاة: oui	/non

*Insatisfaction des soins prodigués عدم الرضا على الخدمات المقدمة: oui	/non

*Lieu de travail surpeuplé اكتظاظ: oui	/non

*Je ne sais pas vraimentلا اعرف : oui	/ non

*Charge de travail : Oui	/ non

*Partage des activités : oui	/ non

*Autres raisons :………………….

- **Horaire de violence :** توقيت الحصة

Jour	/ Après-midi	/	Nuit

-**Lieu de violence:** Unité d’hospitalisation	/Unité d’urgences	/ Unité de consultation externe	/Autres..............................................

-**Poursuite juridique** تتبعات قضائية:

Oui	/	Non

- **Si pas de poursuite juridique les raisons :**

*Avoir peur الخوف: oui	/ non

* c’est inutile دون جدوى: oui	/ non

* Je ne sais pas les procédures عدم دراية:

oui	/ non

*Avoir honte التحفظ: oui	/	non

*Pas important غير مهم: oui	/	non

*Aucune raison بدون سبب: oui	/	non

*Autres raisons……………………………..

-**Attitude immédiate** après **la violence ردة الفعل الفورية:**

*Tentative de gestion de la colère de la personne تهدئة: Oui	/	non

*Lui demander d’arrêter طلب التوقف: oui	/ non

*Appel de la sécurité de l'hôpital الاتصال باعوان الحراسة: oui	/non

*Appel à l'aide de la police الاتصال بالشرطة:

oui	/	non

*Riposte (violence de ma part) الدفاع عن النفس: oui	/	non

*Appel à l'aide de collègues الاستعانة بزميل:

oui	/	non

*Appel du supérieur au travail الاستعانة بمسؤول: oui	/ non

*Quitter le poste de travail المغادرة: oui	/non

*Ne rien faire بدون ردة فعل:oui	/ non

*Autres :……………………………….

- **Les émotions ressenties :**

*Colèreغضب  : oui	/ non

*Frustration ذعر: oui	/	non

*Peur خوف: oui	/ non

*Déception احباط : oui	/	non

*Tristesse حزن: oui	/ non

*Injustice ظلم: oui	/	non

*Intensité de toute émotion مشاعر مختلطة و قوية: oui	/ non

- **Les Mesures prises par les autorités sanitaires الإجراءات المتخذة اداريا:**

*Déclaration à l’administration par écrit : oui	/	non

*Rien لا شيء: oui	/	non

*Sanction du contrevenant مجازاة المعنف: oui	/ non

*Améliorer les mesures de sécurité تحسين إجراءات الحماية: oui	/	non

*Soutien moral الإحاطة النفسية: oui	/	non

*autres……………………………………

- **Les Conséquences** المخلفات: oui	/ non

-Si oui : Psychologiques	/Physiques

**-Si Conséquences Physique جسدية:**

*Douleur اوجاع: oui	/	non

Intensité (de 1 à 10) :………

*Cicatrices خدوش: oui	/ non

*Blessures جروح: oui	/ non

*Fractures كسور: oui	/	non

*Incapacités سقوط: oui	/ non

*Repos récupérateur راحة مرضية: oui	/	non

*Hospitalisation إقامة بمستشفى: oui	/	non

*Usage de médicaments ادوية: oui	/	non

*Autres…………………………..

**- Si Conséquences psychologiques نفسية:**

*Pensez à changer de lieu de travail التفكير في تغيير مكان لعمل:

oui	/ non

*Penser à changer de travail التفكير في تغيير العمل: oui	/	non

*Prévoi de quitter le pays الهجرة: oui	/ non

*Réflexion sur la performance التشكيك في الكفاءة: oui	/	non

*Se sentir en manque de sécurité au travail قلة الامان:

oui	/ non

* Avez-vous consulté un professionnel de santé mentale (psychiatre, psychologue ou autre professionnel qualifié) pour l'un des problèmes suivants après cet incident ?

*Trouble de stress post-traumatique :

Oui □ □ Non

*Anxiété : □ Oui □ Non

*Dépression : □ Oui □ Non

*Évoquer toujours la scène التفكير الدائم في الحادثة: oui	/ non

*Etat de stress post-traumatique ضغط مرضي: oui	/ non

*Troubles de sommeil اضطراب نوم: oui	non

*Eviction de travail عدم الذهاب للعمل: oui	non

*Mauvaise perspective de l’avenir غياب الأمل:

oui	/	non

*Retentissement sur la vie sociale انعكاسات على الحياة الاجتماعية:

Oui	/	non

*Être super alerte اليقظة الدائمة: oui	/	non

Avoir l'impression que tout ce que vous faites est un effort الشعور بأن كل عمل يتطلب مني مجهود كبير: oui	/	non

*Affecter la façon dont vous traitez avec collègues à l'avenir انعكاسات على علاقتي بزملائي: oui	/	non

Autres…………………………………………
